# Ketamine’s Role in Neuroinflammation and Neuroprotection Across Neurological and Psychiatric Disorders: A Narrative Review

**DOI:** 10.3390/ph18091298

**Published:** 2025-08-29

**Authors:** Gustavo N. Silva, Virna G. A. Brandão, Kenneth Blum, Kai-Uwe Lewandrowski, Rossano K. A. Fiorelli

**Affiliations:** 1 Department of Anesthesiology, Gaffrée e Guinle Universitary Hospital (EBSERH), Federal University of the State of Rio de Janeiro (UNIRIO), Rio de Janeiro 22290-240, RJ, Brazil; 2 Division of Addiction Research & Education, Center for Sports, Exercise & Mental Health, Western University of Health Sciences, Pomona, CA 91766, USA; 3 Center for Advanced Spine Care of Southern Arizona, Tucson, AZ 85712, USA; 4 Surgical Institute of Tucson, Tucson, AZ 85711, USA; 5 Department of General and Specialized Surgery, Gaffrée e Guinle Universitary Hospital (EBSERH), Federal University of the State of Rio de Janeiro (UNIRIO), Rio de Janeiro 22290-240, RJ, Brazil

**Keywords:** neuroprotection, neuroinflammation, NMDA receptors, brain injury, ketamine

## Abstract

Ketamine, a widely used anesthetic with emerging evidence suggesting neuroprotective and anti-inflammatory properties across various neurological disorders, is recognized for its NMDA receptor antagonism. It has been postulated to play a role in neuroprotection, due to its anti-inflammatory properties, and decrease microglial activation, as well as cytokines TNF and IL-6. Despite its established role, the extent of ketamine’s effects on neuroinflammation and neuroprotection remains to be fully elucidated. Here, we conducted a narrative review synthesizing current knowledge on ketamine’s operating mechanisms, including its modulation of synaptic plasticity, excitotoxicity, and cytokine release, alongside its therapeutic applications in traumatic brain injury, neurodegenerative diseases, psychiatric disorders, and pain management. For this narrative review, we searched the Medline, Embase, Scopus, Web of Science, and PubMed databases. Our findings indicate that ketamine reduces excitotoxicity and inflammation, which may contribute to neuroprotection in acute neurological injuries. These insights underscore ketamine’s potential as an adjunctive neuroprotective agent, warranting further clinical investigation to optimize its therapeutic utility across neurological and psychiatric contexts.

## 1. Introduction

The first human study involving ketamine, a derivative of phencyclidine, was carried out in the 1960s. The compound induced significant analgesia associated with a distinct state of altered consciousness (“dissociation”) [1]. After half a century, this drug and its enantiomers continue to occupy a unique position in the treatment of various pathologies in areas such as anesthesiology, intensive care medicine, and psychiatry. In the latter field, patients with treatment-resistant depression and coexisting pain showed reduced TNF-α and IL-6 levels, as well as antidepressant and analgesic effects. These changes appear to result from the synchronization between the immune response system (IRS) and the compensatory immunoregulatory reflex system (CIRS). Subanesthetic doses of ketamine may be effective and even lead to clinical improvement after six infusions. This is achieved through the modulation of NMDA receptors (NMDARs) in the cerebral cortex and hippocampus [2]. However, ketamine increases the risk of nausea, vomiting, headache, hallucinations, and dizziness compared to placebo in surgical models [3].

Although research into the effects of ketamine on functional outcomes and mortality in patients with severe brain injuries is quite limited, it can inhibit cortical depolarization, an electrophysiological event that can trigger secondary brain damage after a primary brain injury [4]. Potential advantages for patients with severe brain lesions can be attributed to a reduction in excitotoxicity by decreasing extrasynaptic stimulation of neurotoxic NMDARs and presynaptic glutamate release, resulting in a reduction in calcium-mediated cell death processes. Ketamine also reduces the release of pro-inflammatory cytokines, including IL-8 and TNF-α from microglial cells. In cerebral microvasculature, it can reduce microthrombosis by inducing platelet aggregation and maintain or increase cerebral blood flow through cerebral vasodilation. Its therapeutic effects may have a positive impact on dendritic spine density regulation, potentially leading to the emergence of new neuronal synapses during the recovery period [5].

NMDARs are members of the glutamate receptor family, ligand-gated channels that coexist with other glutamate systems, and they represent a highly complex neurotransmitter system. It is distributed at different levels of the CNS (pre-, post- and extrasynaptic and different brain regions), and the biochemical organization of its receptors into four subunits adds to the complexity of the system. These receptors are divided into two distinct groups: metabotropic receptors (mGluRs) and ionotropic receptors (iGluRs) [6]. Glutamate-mediated excitotoxicity is a crucial pathological process involved in neurodegenerative disorders. Targeting the role of these receptors holds therapeutic promise in chronic conditions characterized by progressive neuronal loss, which are globally increasing and abruptly generating economic and social burdens [7].

Ketamine works by antagonizing NMDARs. This results in the release of glutamate, activating other receptors that increase synaptogenesis and improve signaling via neurotrophic factors in brain regions. It also restores dopamine transmission, which helps to reduce symptoms such as anhedonia (loss of pleasure), although it can cause psychotic effects due to the release of dopamine in certain areas of the brain. NMDARs act as amplifiers and modulators of excitatory signals in the dorsal horn of the spinal cord and in certain specialized peripheral circuits. They interact with μ-opioid receptors (MORs), with such interaction reducing glutamate release. Chronic activation of NMDARs contributes to opioid-induced tolerance and hyperalgesia. In the mesolimbic (reward) pathway, NMDARs modulate synaptic plasticity in the ventral tegmental area (VTA) and nucleus accumbens. These regions are critical for reinforcing effects of opioids and influence addiction and withdrawal circuits. Additionally, modulation of GABAergic interneurons in VTA can lead to disinhibition of dopaminergic neurons [8].

In an intensive setting, a study of children with severe traumatic brain injury found that, following a protocol based on guidelines, ketamine was associated with a reduction in intracranial pressure under sedation and an increase in cerebral perfusion pressure above the threshold [9]. In animal models, esketamine at a subanesthetic dose demonstrated protection against neurological deficits induced by traumatic brain injury and cerebral edema, improving oxidative stress, neuronal cell death, and apoptosis [10].

Significant gaps exist in the understanding of neuroinflammation and the effect of ketamine on post-trauma secondary brain processes resulting from glutamate excitotoxicity. Our study, therefore, addresses the primary mechanisms involved in neuroprotection and provides some therapeutic evidence in the context of neurological and psychiatric disorders.

## 2. Methods

This study is a narrative review about the use of ketamine in various clinical contexts, including traumatic brain injuries, subarachnoid hemorrhage, acute and chronic pain, depression, drug detoxification, seizures, and anesthetic requirements. Our study reviews the potential mechanisms underlying the neuroprotective effects of ketamine. To this end, the authors searched and selected papers from three widely used health databases: PubMed, MEDLINE, and EMBASE. The search was conducted from October 2024 to June 2025 based on specific health sciences descriptors (DeCSs)—“Brain injury” AND “Intracranial pressure” AND “ketamine” AND “Neuroprotection” AND “Neuroinflammation”—in various combinations. At a certain point, the descriptors were crossed with each other, and some filters were added as inclusion criteria: studies from 2010 to 2024, free full texts, clinical trials, systematic reviews, and observational studies. Case reports and letters were not assessed. Subsequently, 424 articles were selected for abstract reading, of which 32 matched the research criteria. Additionally, 37 supplementary articles were included in the study through a manual search.

For didactic purposes, this article is divided into sections, firstly providing an overview of NMDARs, before discussing the protective effects of ketamine on neurodegenerative diseases, its role in neuroprotection in some clinical situations, ketamine in addiction and psychiatric disorders, and ketamine’s therapeutic effects as an adjuvant drug in neuroanesthesia.

## 3. Results

### 3.1. An Overview of NMDRAs

**Figure 1:**
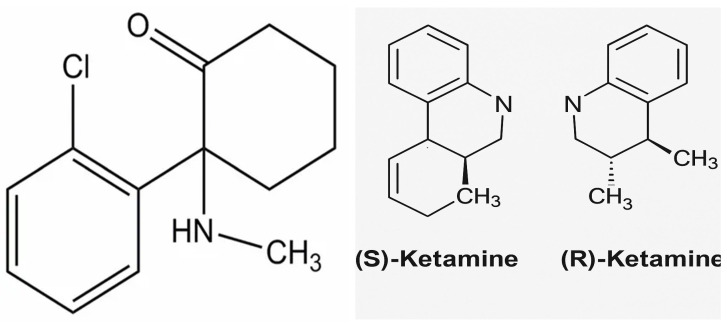
Racemic ketamine and its enantiomers structure. Ketamine exists as S(+) and R(−) isomers, which are commonly marketed as a racemic mixture. The S(+) isomer of ketamine has a higher affinity for the binding site on N-methyl-D-aspartate (NMDA) receptors and produces anesthetic potency three to four times greater than the R(−) isomer.

Ketamine exists as S(+) and R(−) isomers and is commonly marketed as a racemic mixture (Figure 1). N-methyl-D-aspartate receptors (NMDARs) are members of the glutamate receptor family, divided into metabotropic and ionotropic receptors (iGluRs). Metabotropic glutamate receptors are G protein-coupled receptors (GPCRs) that are not restricted to the glutamatergic synapse and glial cells, including astrocytes, oligodendrocytes, and microglia. They have a slow activation rate and are responsible for delaying synaptic responses, which can lead to integration of temporally dispersed signals. On the other hand, iGluRs are responsible for rapid excitatory synaptic transmission. Their fast kinetics allow them to respond to a brief release of glutamate into the synaptic cleft. Among the three main classes of iGluRs, α-amino-3-hydroxy-5-methylisoxazole-4-proprionic acid receptors (AMPARs) and NMDARs are predominantly postsynaptic, whereas kainate receptors (KARs) are pre- or extrasynaptic and appear to play a modulatory role in synaptic transmission [6].

In mammals, NMDARs exist as several subtypes that differ in their molecular, anatomical, and signaling properties. They are considered the primary pathway through which ketamine acts, though they are not the only pathway. When activated, the glutamate-activated ion channel allows Ca^2+^ and Na^+^ to enter and K^+^ to exit. Ketamine binds inside the channel, at an allosteric site that is only accessible when the channel is opened. This is why ketamine is called a use-dependent blocker. Ketamine reduces the influx of calcium and sodium by blocking the channel, thereby decreasing neuronal excitability and modulating synaptic plasticity. This action involves anesthesia and analgesia that act on the dorsal horn of the spinal cord, thereby reducing nociceptive transmission, mainly from C and Aδ fibers. Additionally, it interferes with the wind-up phenomenon and N-methyl-D-aspartate (NMDA)-mediated central sensitization. Antidepressant effects occur through action on GABAergic interneurons and the disinhibition of glutamatergic neurons. These effects occur rapidly, within hours, and last for few days, even after the drug’s direct action has ended. Dissociative and psychomimetic effects, which involve reduced NMDA transmission in the cortex and hippocampus, are common. These effects alter sensory integration and perception of reality, leading to the typical subjective effects [11].

Excitatory neurotransmission and cell signaling mediated by NMDARs are essential for brain development. The transmission of glutamate and the activation of its ionotropic receptors are fundamental mechanisms by which neurons control their synaptic functions modulation and information processing. The N-methyl-D-aspartate receptor is unique among all ligand-dependent channels, requiring two ligands—glutamate and glycine—for activation. These receptors function as heterotetrameric ion channels, with channel opening dependent on the binding of glycine and glutamate to the extracellular ligand-binding domains of the GluN1 and GluN2 subunits, respectively. The opening of NMDAR channels and the subsequent relief of Mg^2+^ blockade by membrane depolarization can lead to an influx of sodium and calcium, which is associated with higher-order brain functions, including learning and memory. Its dysfunction has been implicated in various neurological diseases and disorders, such as schizophrenia, Alzheimer’s disease, depression, and acute neuronal damage resulting from stroke or traumatic brain injury [12].

Furthermore, their activity is associated with the modulation of the cell cycle. Overactivation of NMDA receptors, for example, has been demonstrated to cause excessive calcium influx, leading to the expression of key elements that regulate and potentially disrupt the cell cycle [13]. Recent findings indicate that mutations in genes encoding various NMDAR subunits are associated with neurological diseases. Therefore, the functional analysis of these mutant receptors will help to better characterize the role of each subunit in controlling receptor signaling in brain circuits and will drive the development of therapeutic drugs for neurodevelopmental disorders as new generations of positive allosteric modulators [14].

### 3.2. Protective Effects on Neurodegenerative Diseases

Animal studies have shown promising methods with the use of ketamine, alone or in combination, to increase memory consolidation and cell proliferation, thus improving cognitive functions [15,16]. Early studies show that ketamine may be effective in treating PD-associated depression and reducing levodopa-induced dyskinesia (LID). However, positive RCTs are needed to change clinical practice. It is not approved for the treatment of motor symptoms of PD and is only used in clinical trials. In Alzheimer’s disease (AD), there is biological rationale and case reports, but there is no robust clinical evidence of sustained benefit, so it remains experimental. Esketamine is only approved for treatment-resistant depression outside the context of dementia. However, the tools and therapeutic approaches to prevent or reverse synaptic damage are not entirely clear. It is necessary to understand recent advances in molecular pathways that impair synapses in neurodegenerative diseases and their effects on neuroinflammation.

For example, a study using human GABAergic projection neurons derived from human inducible pluripotent stem cells and embryonic stem cells in vitro tested the effects of ketamine on growth cones and synapses in developing GABAergic neurons, evaluating the glycogen synthase kinase-3 (GSK-3) and histone deacetylase 6 (HDAC6) pathways. Exposure to ketamine impaired growth cone formation, synaptogenesis, dendritic development, and maturation through mediated activation of GABAergic neurons. The identification of this neurotoxic pathway suggests that microtubule acetation could serve as a potential target for mitigating the toxic effects of ketamine on neuronal development of striatal GABAergic projections in both mature and early developing neurons [17]. Studies are progressing in search of solutions aimed at restoring synaptic integrity, preventing synaptotoxicity, modulating neuronal activity, and targeting immune signaling.

#### 3.2.1. Parkinson’s Disease

Parkinson’s disease involves the gradual degeneration of dopaminergic neurons within the substantia nigra, leading to a reduction in dopamine release in the striatum that results in movement disorders such as tremors, rigidity, and bradykinesia, as well as cognitive impairments such as dementia. Additionally, it manifests as mental symptoms like depression and anxiety, along with disruptions in sleep patterns and bowel functions [18]. Low-dose subanesthetic ketamine treatment in rodents has indicated the significance of its metabolites, hydroxorketamine and norketamine, for acute antidysetic and antiparkinsonian activities. Opioid tone, especially in the direct striatum/D1, is linked to the genesis of LID. Ketamine affects the opioid system, and there is evidence of the functional affinity of its metabolites for μ/κ (and δ) receptors in cell preparations [19].

In LID, the role of opioids in ketamine’s action appears to be “modulatory”. Blocking opioid receptors with naloxone did not abolish ketamine’s acute antidyskinetic effect; rather, it enhanced its antiparkinsonian effect, improving akinesia. Therefore, the opioid pathway, mediated by NMDA, acts as a modulator, and opioid antagonism may help with certain motor aspects, at least in preclinical models. However, controlled clinical trials in PD/LID are still needed to determine the precise role of opioid receptors in this context. This demonstrates the involvement of the opioid system of Parkinson’s disease treatment and levodopa-induced dyskinesia [19].

Ketamine reversed short-term memory impairment and depressive behaviors in rats with bilateral lesions in substantia nigra compacta, indicating a promising profile for its use in patients with PD [20]. Another NMDAR antagonist, agmatine, a natural polyamine derived from larginine, has neuroprotective properties in PD. Its mechanisms of action include antioxidant, anti-apoptotic and anti-inflammatory effects, as well as protection of the blood–brain barrier. In animal models using rotenone, agmatine has demonstrated the ability to minimize oxidative damage, alleviate motor deficiencies, reduce loss, and increase the expression of plasticity-related proteins in the brain. It also has anti-inflammatory effects, reducing the levels of pro-inflammatory cytokines elevated in PD, which could slow down the progression of the disease and improve quality of life [21].

#### 3.2.2. Alzheimer’s Disease

Advances in public health have resulted in an aging population, creating an environment conducive to an increase in Alzheimer’s disease (AD) prevalence. Positron emission tomography markers to identify patterns of neuritic plaque deposition (amyloid) and neurofibrillary tangle formation, together with histochemical observations of neuroinflammation, neuronal synapses loss, and brain atrophy, have proved important in understanding the pathological events that define AD [22].

The p38MAPK signaling pathway plays an important role in regulating memory and learning processes, and studies have shown that high levels of phosphorylated p38 MAPK expression in peripheral blood are associated with AD and PD [23,24]. Low concentrations of ketamine promoted neuronal survival and reduced apoptosis in cultured hippocampal neurons from rat fetuses in vitro, suggesting that selective degradation of phosphorylated p38 MAPK may offer a promising approach for the treatment of AD [25]. However, it remains unclear whether additional signaling pathways contribute to the neuroprotective or neurotoxic effects of ketamine, necessitating further research to provide a more definitive understanding of ketamine’s impact on the developing brain.

Ketamine has proven successful in treating psychiatric symptoms, particularly treatment-resistant depression. Recent clinical studies suggest that ketamine may have potential for providing neuroprotection and reducing psychiatric symptoms, especially depression, apathy, agitation, and mild psychosis, in patients with Alzheimer’s disease (AD). This conclusion is supported by both neurobiological mechanisms and specific clinical observations. AD is characterized by glutamatergic dysfunction, which involves excessive activation of NMDA receptors by extracellular glutamate. This leads to excitotoxicity and synaptic dysfunction. Ketamine reduces excessive calcium influx into neurons, thereby protecting against damage and stabilizing transmission. By activating AMPA pathways, ketamine increases the release of BDNF (brain-derived neurotrophic factor), which stimulates plasticity. It signals the mTOR pathway, which stimulates synaptic protein synthesis, the formation of new dendritic spines, and the restoration of corticolimbic circuits. Additionally, ketamine can inhibit microglial activation and reduce pro-inflammatory cytokines (IL-6 and TNF-α), which may reduce neurotoxicity and improve neuropsychiatric symptoms. These effects help restore networks involved in mood, motivation, and emotional response, all of which are impaired in AD [26].

A study in mice provides new insights into the antidepressant mechanism of ketamine, revealing that astrocyte pyroptosis in the hippocampus is reversed by improving the glymphatic pathway—a potential therapeutic target for depression [27].

#### 3.2.3. Multiple Sclerosis

Multiple sclerosis (MS), an autoimmune and degenerative disease that affects the central nervous system, shows long-term reductions in fatigue-related symptoms after low-dose ketamine infusions [28,29].

Research indicates a correlation between demyelination and microglial activation in the corpus callosum of mice treated with cuprizone, a compound used to induce ME-like demyelination. Ketamine was found to enhance demyelination and microglial activation through the TrKB stimulation, a tyrosine protein kinase receptor that acts as a brain-derived neurotrophic factor and other neurotrophic receptors. Additionally, ketamine partially restored the diversity of intestinal microbiota in these mice and facilitated a reduction in lactic acid levels in their stool [30]. Thus, it is plausible that gut microbiome–microglia integration may play a role in ketamine’s effects in ME.

Ketamine has demonstrated its ability to mitigate progression of experimental autoimmune encephalomyelitis in mice, a widely utilized model for multiple sclerosis. It alleviated body weight loss, enhanced clinical scores, and significantly decreased pathological scores, promoting microglial activation and preserving blood–brain barrier integrity in the spinal cord [31]

There are no robust clinical trials testing the use of ketamine for motor symptoms or MS progression. However, it may be beneficial for treating refractory neuropathic pain. Some small studies have shown that subanesthetic infusion of ketamine significantly reduces pain, which is a typical effect in central control. In cases of treatment-resistant depression, there are isolated reports of rapid improvement in mood and fatigue, similar to those observed in patients with other diseases. No human studies have demonstrated an impact on disease progression, reduction in outbreaks, or preservation of myelin [28,29].

### 3.3. Role in Acute Neuroinflammatory Disorders

Ketamine’s safety profile and fleeting action make it a reliable tool in pre-hospital environments. It maintains a hemodynamic profile by preventing hypotension, reducing fluid use, and preserving spontaneous ventilation, all while also offering the potential to optimize analgesia and sedation in the management of ventilated patients with traumatic brain injury [31]. In perinfarct tissues, a study carried out on a mouse model showed that ketamine effectively reduced intracellular Ca^2+^ accumulation in adjacent brain lesion areas after a wave of depolarization, attenuating the harmful effects on the brain under metabolic impairment conditions [32].

A protective effect of ketamine may occur after traumatic brain injury (TBI), probably due to the suppression of the onset of widespread depolarization. This is an abnormal propagation of electrical activity that can be detected by electroencephalography (EEG) or internal measurement with suitable electrodes. It is associated with poor outcomes in patients with TBI after brain damage. No differences in ICP were detected between patients who received ketamine and the control group. None of the patients showed pathological ICP. Ketamine was found to prevent cough reflexes and show reduced ICP values in the short term and increased values in the long term without evidence of threat. In the pediatric population, a decrease in ICP median of 7.8 mmHg was observed two minutes after a ketamine bolus. Additionally, ketamine increases cerebral perfusion pressure and consequently decreases the dosage of vasopressors needed to counteract the effects of opioid-based sedatives. Due to its neuromodulatory properties, ketamine may be a safe drug to use alone or in combination with other sedatives in patients with moderate to severe spinal cord injury (SCI) who require mechanical ventilation [9,33,34].

Early administration has demonstrated effectiveness with a response rate in refractory status epilepticus patients who are unresponsive to anticonvulsants for whom mechanical ventilation is contraindicated due to comorbid conditions [35]. In neonatal and pediatric populations, a retrospective cohort study of patients admitted to intensive care units showed that ketamine administration was associated with minimal adverse events, and seizures ceased with its use [36]. Although effective in the early stages of epileptic seizures, GABAergic drugs, such as benzodiazepines and phenobarbital, have limited efficacy during prolonged seizure activity due to receptor trafficking, which leads to internalization within the intrasynaptic membrane. On the other hand, NMDA receptors are positively regulated, which provides a pathophysiological justification for their early administration, thus providing neuroprotection and reducing dependence on vasopressors [37].

Ketamine could be considered a potential option for symptomatic and restorative therapies in neuromyelitis optica spectrum disorders, particularly in cases involving spinal cord lesions in cervical and thoracic segments, including around the central canal and adjacent gray matter in the dorsal and ventral horns. In early active lesions, with reducing neuroinflammation emphasis, ketamine may offer relief by blocking NMDA receptors to inhibit long-term potentiation in C-fiber synapses responsible for neuropathic pain [38,39].

Coronavirus disease 2019 (COVID-19), responsible for severe acute respiratory syndrome coronavirus 2 (SARS-CoV-2), has been associated with neurological and neuropsychiatric complications, including anxiety, depression, mood disorders, and psychosis. The hypothesis is that these symptoms result from an excessive systemic inflammatory response, which manifests itself as viral sepsis, immune-mediated mechanisms, or direct damage to the cerebral vasculature induced by the virus. Immune dysregulation, such as defective antigen presentation caused by interleukin 6 (IL-6) or an IL-1β-mediated hemophagocytosis-like syndrome, may play a role in depression. Dopaminergic or glutamatergic drugs, such as ketamine, can help prevent serious alterations in neurotransmitter metabolism. Ketamine has been shown to downregulate several inflammatory cytokines, including IL-6, while changes in cytokine levels correlate significantly with symptom improvement [40].

### 3.4. Neuromodulation in Psychiatric Disorders

The literature highlights the antidepressant and antisuicidal effects of ketamine in unipolar/bipolar depression, as well as in treatment-resistant depression, with repeated doses extending the duration of efficacy. In several studies, ketamine possesses short-term antisuicidal properties, independent of its impact on depressive symptoms [41]. The mechanisms underlying its effects involve both NMDAR antagonism and non-NMDAR pathways. The resulting increase in excitatory neurotransmission and the induction of neurotrophic factors linked to antidepressant effects have long been considered a fundamental mechanistic model [42].

Brain-derived neurotrophic factor (BDNF) and its receptor, tropomyosin receptor kinase B (Trk), are believed to be essential for sustained remodeling of the affect circuit. Polymorphisms in the genes encoding TrkB and NMDAR are known to modulate these therapeutic results [43]. A clinical study showed that adolescents diagnosed with major depressive disorder who received a single intravenous infusion of ketamine (0.5 mg/kg over 40 min) showed a significant reduction in depressive symptoms 24 h after infusion compared to midazolam. The biological effects of ketamine can be attributed to increased glutamatergic signaling, mediated by NMDA antagonism of prefrontal GABAergic interneurons and stimulation of AMPA receptors through increased release of glutamate or ketamine metabolites. The teenage brain represents a distinct pharmacological substrate, characterized by the continuous maturation of monoaminergic, glutamatergic, and GABAergic systems, requiring consideration of the developmental pharmacological context when evaluating new therapies [44].

S- and R-ketamine have differential effects on inflammation-induced depression using a lipopolysaccharide (LPS)-induced mouse model. This condition is associated with neuroinflammation, as evidenced by elevated levels of pro-inflammatory markers such as TNF-α and IL-1β. S-ketamine significantly alleviated depressive behaviors and reduced pro-inflammatory factor levels in the medial prefrontal cortex by suppressing pro-inflammatory gene expression [45].

In addition to reducing suicidal ideation, it may be a potential therapeutic option for patients with treatment-resistant anxiety pathologies, particularly obsessive–compulsive disorder and post-traumatic stress. Patients with eating disorders may experience remission of severe anorexia nervosa. When used alone or in conjunction with other therapies, it has been shown to be effective in reducing alcohol and substance use, decreasing cravings and increasing motivation, especially when combined with motivational enhancement treatment [46].

Participants who received selective serotonin reuptake inhibitors were more likely to respond to ketamine treatment. Case reports, non-randomized studies, and randomized studies have all consistently reported immediate improvements in symptoms within 24 h. However, long-term improvements, which were sometimes sustained for two to eight weeks, were more consistently reported in case reports and non-randomized studies than in randomized studies [47].

Unlike cocaine, the long-term effects of ketamine on mesolimbic reward circuits and behavior do not indicate the induction of drug-adaptive synaptic plasticity, long-term locomotor sensitization, or uncontrolled self-administration. Ketamine has rewarding and reinforcing properties because it indirectly acts on the dopamine system through the effects of local GABA neurons on circuits. The absence of adaptive synaptic plasticity indicates that ketamine dependence is limited by its pharmacology [48].

Concerns persist about the addictive potential of ketamine for long-term depression treatment. Despite the heterogeneity of study designs and methods of assessing results, this study emphasizes the relative safety of ketamine treatment for adult patients. It also highlights the importance of physician-supervised administration, vigilant monitoring, and careful dosing [49]. Table 1 points to behavioral parameters related to the use of ketamine treatment in neurological disorders that support ketamine’s neuroprotection.

Patients with refractory chronic pain who were treated with (R,S)-ketamine showed no differences in pain, anxiety, or ketamine dosage according to gender or age. The baseline inflammatory state may be more pronounced in men with neuropathic pain than in women with fibromyalgia. Ketamine exerts a strong immediate anti-inflammatory effect with the first dose; however, there is little remaining inflammation and less pain relief with subsequent administrations. In elderly patients and men aged ≥50 years, who have an even greater inflammatory state and higher IL-6 concentrations, ketamine prevents a drop in analgesia with just one administration [50].

Despite its association with transient and dose-dependent psychotomimetic and cardiovascular side effects, it is used to treat neuropathic, acute, and chronic postoperative pain. It interacts with α-amino-3-hydroxy-5-methyl-4-isoxazolepropionic acid (AMPA) receptors, as well as opioid, cholinergic, catecholaminergic, and hyperpolarization-activated cyclic nucleotide-gated (HCN) receptors. Due to its downstream signaling mechanisms, it is an option for individuals with depression and concomitant acute or chronic pain. Mood disorders and pain often occur together, share neurobiological mechanisms, and are difficult to treat [51].

### 3.5. Therapeutic Effects as an Adjuvant Drug in Neuroanesthesia

**Figure 2:**
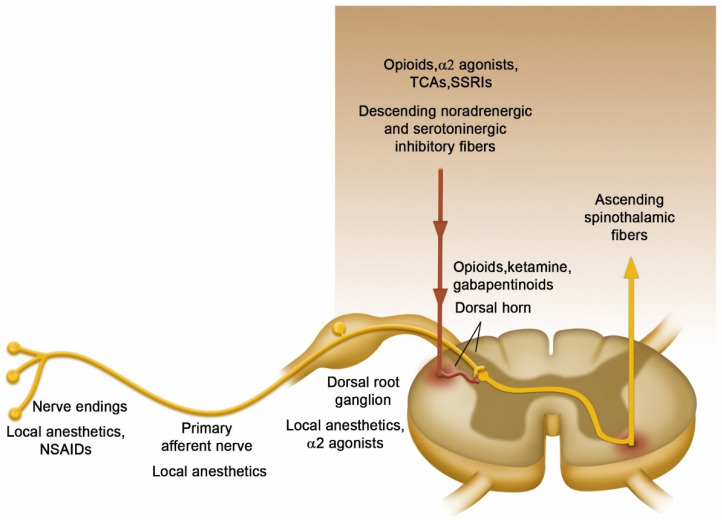
Pain processing involves molecular and neural modulation in several areas, including the peripheral nociceptor, the nerve, the dorsal root ganglion, the dorsal horn of the spinal cord, the brain, and the brainstem. The NMDA receptor mainly affects pain modulation in the dorsal horn of the spinal cord. The figure illustrates the possible anatomical sites of the pain signaling pathway.

Therapeutic interventions can significantly reduce inflammation in response to infection, surgery, or trauma, thereby improving patient outcomes and reducing health-related costs [52]. Protective approaches in medical treatment, such as dexmedetomidine, ketamine, opioid-sparing techniques, ischemic and pharmacological preconditioning interventions, strategies to maintain endothelial glycocalyx integrity, and peripheral/neuroaxis blocks represent therapeutic pathways that decrease cellular damage and surgical inflammatory response (Figure 2) [53,54,55,56].

The sympathomimetic profile of ketamine, together with its neuroprotective effect, may be advantageous over other sedative and analgesic drugs used in patients with traumatic brain injury who are at risk of experiencing a reduction in blood pressure, which could exacerbate secondary neurological injury. A study of adult patients with severe traumatic brain injury randomized to receive an infusion of either ketamine at 3 mg/kg/h or normal saline showed that, during the first four to six hours, patients in the ketamine group had significantly higher blood pressure and CPP levels than those in the control group. However, this effect was not sustained beyond six hours, and a significant reduction in ICP was observed only briefly, between the fourth and sixth hours. The control group required more vasopressors; however, there was no difference in neurological outcome at three months [57]. After its administration, the respiratory impulse and the protective reflexes of the upper airways are generally preserved, allowing spontaneous ventilation to be maintained. However, high doses that have an anesthetic effect or are used to control convulsive or disseminated depolarization (>1 mg/kg) carry the risk of suppression and thalamo-cortical dissociation. Clinical data and case studies support the therapeutic effect of ketamine in suppressing disseminated depolarization after brain injury. This phenomenon is believed to be an important mechanism of secondary brain injury and late cerebral ischemia. Ketamine also causes pulmonary vasodilation and bronchodilation, resulting in an increase in mean arterial pressure and heart rate. Its inhibitory effects on the gastrointestinal system are minimal, and it may also have antiplatelet activity [58,59,60].

Sedatives are often used to control intracranial pressure, reduce cerebral metabolism, enable other treatments such as mechanical ventilation or targeted temperature control, and control sympathetic hyperactivity. However, the U.S. Food and Drug Administration (FDA) has issued a precaution regarding the use of ketamine in patients with elevated intracranial pressure (ICP) prior to anesthesia, which discourages its use in patients with traumatic brain injury (TBI). Additionally, no randomized clinical trial (RCT) has shown that ketamine is completely safe for these patients. The Brain Injury and Ketamine (BIKe) study is investigating the safety of using ketamine as an adjunct to a standard sedation regimen in patients with TBI [61].

Cerebral autoregulation involves adjustments in vascular tone sensitive to variations in systemic blood pressure. These changes are thought to be regulated myogenically or endothelium-dependently in response to stress in the vasculature; neurogenically, by the autonomic nervous system and neurons; and, to some extent, metabolically. Most anesthetic agents reduce the brain’s overall global oxygen metabolism, while volatile anesthetics, nitrous oxide, and ketamine increase cerebral blood flow. However, other intravenous agents, such as propofol and midazolam, reduce it. These changes in physiological parameters affect various endogenous mechanisms, including cerebral autoregulation, vasomotor reactivity, and neurovascular coupling [62]. Randomized study evaluating the effects of a propofol–ketamine mixture for anesthesia induction and maintenance in patients undergoing emergency decompressive craniectomy for traumatic brain injury (TBI) showed improved hemodynamic stability and reduced need for vasopressors, with cerebral relaxation similar to those of the propofol-only group. On the third postoperative day, glial fibrillary acidic protein (GFAP) was lower in the ketofol group (3.31 ± 0.43 ng/mL) than in the propofol group (3.41 ± 0.17 ng/mL; *p* = 0.01). GFAP is an essential component of astrocytic intermediate filaments that confer structural stability. It plays an important role in cell structure and is an indicator of the response to traumatic brain injuries, strokes, and neurodegenerative diseases [63].

Cortical spreading depolarization (CSD) can cause neurological deterioration after evacuation of the subdural hematoma. A report of three isolated cases in which patients were treated with ketamine empirically indicated that it can reverse neurological deficits not explained by neuroimaging or electroencephalography, such as seizures [64]. Esketamine can reduce acute postoperative pain in various contexts. However, in a study of vestibular schwannoma resection with total intravenous anesthesia (TIVA) using propofol/remifentanil, the effects of esketamine in low intraoperative doses (0.2 mg/kg) were insufficient to reduce acute pain after surgery, and BIS values increased for at least 30 min after its administration [65]. On the other hand, a systematic review that used electronic databases to search for studies related to the use of ketamine for neuroprotection after cardiac arrest suggested that ketamine may improve neurological outcomes [66]. Additionally, S-ketamine administered via patient-controlled analgesia reduced opioid consumption in a dose-dependent manner following major lumbar fusion surgery. This resulted in a decrease in cumulative oxycodone consumption 24 h later without any additional adverse effects [67]. Despite some controversy, it can be said that ketamine has recently gained significant interest as a treatment for opioid-resistant or “pathological” pain. This includes conditions such as central sensitization with hyperalgesia or allodynia, opioid-induced hyperalgesia, and neuropathic pain.

**Figure 3:**
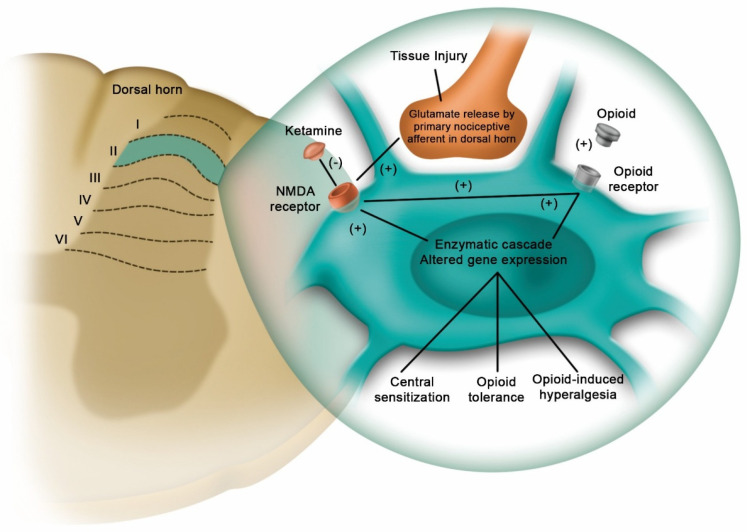
In response to tissue damage or trauma, the primary nociceptive neuron releases glutamate in the dorsal horn of the spinal cord. This glutamate binds to N-methyl-D-aspartate (NMDA) receptors on second-order neurons. Once activated, the NMDA receptor initiates a series of intracellular processes that result in altered behavior, NMDA receptor expression, and neuronal synaptic plasticity. It plays a key role in the development of tolerance and hyperalgesia.

Insufficient analgesia can result in complications, including immobilization, pneumonia, and delayed rehabilitation. Multimodal, opioid-sparing regimens are preferred. A cohort study comparing adult cardiac surgery patients who received a single 0.3 mg/kg dose of ketamine over 30 min with those who did not receive ketamine demonstrated a significant decrease in oral opioid administration (oxycodone). When intravenous opioids were included in the analysis, the results showed a reduction in its use. This may be due to attenuation of the opioid-induced hyperalgesic response by N-methyl-D-aspartate (NMDA) blockade (Figure 3) [68]. Its use as an adjuvant to the local anesthetic bupivacaine was demonstrated in preemptive scalp blocks, focusing on hemodynamics, postoperative pain, and analgesic consumption during supratentorial craniotomy surgery. The study showed that incorporating ketamine into Enhanced Recovery After Surgery (ERAS) strategies in neurosurgery can strengthen them by offering prolonged analgesia, limiting opioid use, and reducing perioperative inflammation [69]. The Table 2 summarizes the applicability of ketamine in neurological clinical practice.

## 4. Conclusions

Ketamine is considered a promising drug with many potential clinical applications. It has a variety of pharmacological effects, ranging from anesthetic induction and maintenance to analgesic and sedative properties, depending on the dosage. Due to its neuroprotective and anti-inflammatory effects, as well as its favorable hemodynamic profile, ketamine is the drug of choice in cases involving opioid tolerance, inflammatory pain, neuropathic pain, depression, or a combination of these factors. However, original studies focused on humans and cancer therapy are still in their infancy. Most current studies use racemic ketamine, but the effect of R-ketamine on racemic ketamine remains unclear. Ketamine reduces excitotoxicity and inflammation, which may contribute to neuroprotection in acute neurological injuries. Despite showing promise in acute situations, tolerance issues limit its long-term use for chronic pain. Its anti-inflammatory effects are attributed to its action on ionotropic glutamate receptors, which play a role in synaptic plasticity, long-term potentiation, and depression.

## Figures and Tables

**Table 1:** Behavioral parameters with ketamine treatment in neurological disorders.

Disorder/Context	Behavioral Parameters Assessed	Observed Effects with Ketamine
Autism Spectrum Disorder (ASD)	– ABC (Aberrant Behavior Checklist) social withdrawal subscale – CGI-I (Clinical Global Impressions scale–Improvement)	Improvement in social withdrawal and overall clinical assessment over ~35 days.
Bipolar depression/treatment-resistant depression	– Depressive symptoms (rapid antidepressant effect) – Suicidal ideation	Rapid reduction in depressive symptoms and suicidal ideation within hours/days, with improvement maintained for up to 3 days after a single dose.
Anxiety and affective comorbidities	– General anxiety, anhedonia, and various affective symptoms	Significant anxiolytic effects, reduced suicidal ideation, and improvement in symptoms such as anhedonia.
Post-traumatic stress disorder (PTSD)	– ‘Freezing’ behavior during re-exposure – Other fear or stress responses	In animal models of PTSD, ketamine worsened ‘freezing’ behavior in animals with extreme behavioral response (EBR), with no changes in BDNF or glucose metabolism.
Prolonged exposure/adolescence	– Locomotion, social behavior, memory, anxiety, motor activity, affective behavior (preclinical)	Changes in locomotion, social behavior, anxiety, depression, memory, and neurotoxic/apoptotic effects in animal models; in human adolescents, mood improvement but risk of cognitive/behavioral impairment.
Prolonged sedation in pediatric patients	– Withdrawal signs (WAT-1) – Behavioral, motor, and cognitive deficits after discontinuation	Withdrawal syndrome, as well as motor and cognitive impairment observed (persistent language deficits detected up to 20 days after hospitalization).
Adult animals (various models)	– Open field behavior, conditioned place preference, immobility, emotional and motor reactivity, conditioned tolerance	Development of tolerance to context-conditioned sedative effects, increased time spent in the center of the open field (low reactivity behavior), no motor impairment; changes in dopaminergic and serotonergic systems.
Postpartum depression in rats	– Forced swim test (immobility, swimming, climbing) – Exploratory activity in the open field – Maternal behavior – Inflammation markers (IL-6, MPO)	Ketamine reversed induced depressive-like behavior (males) and reduced IL-6 and MPO; in females, it reduces MPO in the frontal cortex. It also alters maternal behavior and induces stereotyped behavior with higher prolonged doses.
Neural network/cognition (neural level)	– Short-term memory, dissociative psychoactivity, changes in neuronal synchronization, neural network function	Ketamine affects short-term memory and induces psychedelic effects; it also alters firing patterns and neuronal synchronization, promoting arrhythmic connectivity and potentially modulating cortical networks for therapeutic purposes.
Neuroplastic mechanisms	– Synaptogenesis, neuroplasticity, BDNF expression, mTOR activation, reconsolidation of dysfunctional memories	Stimulation of neuroplasticity via mTOR activation. BDNF increases, promoting brain reconnections and renewal of dysfunctional circuits—the basis for its rapid and profound therapeutic effects.

**Table 2:** The use of ketamine in neurological clinical practice.

Key Points of Clinical Use of Ketamine	Outcomes and Clinical Implications
Mechanisms of action and interaction pathways	Interacts with NMDA receptors, ion channels, including dopamine, serotonin, sigma, opioid and cholinergic receptors, and cyclic nucleotide-gated (HCN) channels activated by hyperpolarization [2]; Lowers levels of TNF-α, IL-6, IFN-γ, IL-10, IL-1β, and IL-4 in patients exhibiting depressive symptoms and associated pain [2]; Increases synaptogenesis and improves signaling through neurotrophic factors in brain regions [8]; Relief of Mg^2+^ blockade by membrane depolarization with an influx of sodium and calcium, which is associated with higher-order brain functions, including learning and memory [12].
Protective effects on neurodegenerative diseases	There is little evidence supporting its use in humans for therapeutic purposes; Animal studies have shown increased memory consolidation and cell proliferation [15,16]; Low-dose use in rodents has indicated activity of its metabolites for acute antidysetic and antiparkinsonian activities [19]; Multiple sclerosis shows long-term reductions in fatigue-related symptoms after low-dose ketamine infusions [28,29];
Role in acute neuroinflammatory disorders	Preventing hypotension, reducing fluid use, and preserving spontaneous ventilation, while also offering the potential to optimize analgesia and sedation in management of ventilated patients with traumatic brain injury [31]; Appears to be a safe drug that can be used alone or in combination with other sedatives in patients with moderate to severe spinal cord injury (SCI) who require mechanical ventilation [33,34]; Ketamine has been shown to downregulate several inflammatory cytokines in viral sepsis, including IL-6 [40].
Addiction and psychiatric disorders	Antidepressant and antisuicidal effects in unipolar and bipolar depression, as well as in treatment-resistant depression, with repeated doses extending the duration of efficacy [41]; Potential therapeutic option for patients with treatment-resistant anxiety disorders, particularly obsessive–compulsive disorder and post-traumatic stress disorder [45]; Acts on dopamine system through the effects of local GABA neurons on circuits. The absence of adaptive synaptic plasticity indicates that ketamine dependence is limited by its pharmacology [48];
Therapeutic effects as an adjuvant drug in neuroanesthesia	Its sympathomimetic profile, together with its neuroprotective effect, may be advantageous over other sedative and analgesic drugs used in patients with traumatic brain injury who are at risk of experiencing a reduction in blood pressure [57]; Therapeutic effect in suppressing disseminated depolarization after brain injury, pulmonary vasodilation and bronchodilation, and increased mean arterial pressure and heart rate [58,59,60]; In patients with elevated intracranial pressure (ICP) before anesthesia, its use in traumatic brain injury (TBI) is not indicated [61]; May improve neurological outcomes after cardiac arrest [66]; S-ketamine administered via patient-controlled analgesia reduced opioid consumption in a dose-dependent manner following major lumbar fusion surgery [67]; Attenuation of the opioid-induced hyperalgesic response by N-methyl-D-aspartate (NMDA) blockade [68]; Incorporating ketamine into ERAS strategies in neurosurgery can strengthen them by offering prolonged analgesia and limiting opioid use [69].

## Data Availability

No new data were created or analyzed in this study. Data sharing is not applicable to this article.
